# Serum Amyloid A Induces Inflammation, Proliferation and Cell Death in Activated Hepatic Stellate Cells

**DOI:** 10.1371/journal.pone.0150893

**Published:** 2016-03-03

**Authors:** Sören V. Siegmund, Monika Schlosser, Frank A. Schildberg, Ekihiro Seki, Samuele De Minicis, Hiroshi Uchinami, Christian Kuntzen, Percy A. Knolle, Christian P. Strassburg, Robert F. Schwabe

**Affiliations:** 1 Department of Medicine, Columbia University, College of Physicians and Surgeons, New York, New York, United States of America; 2 Dept. of Medicine I, University of Bonn, Bonn, Germany; 3 Institutes of Molecular Medicine and Experimental Immunology, University of Bonn, Bonn, Germany; University of Navarra School of Medicine and Center for Applied Medical Research (CIMA), SPAIN

## Abstract

Serum amyloid A (SAA) is an evolutionary highly conserved acute phase protein that is predominantly secreted by hepatocytes. However, its role in liver injury and fibrogenesis has not been elucidated so far. In this study, we determined the effects of SAA on hepatic stellate cells (HSCs), the main fibrogenic cell type of the liver. Serum amyloid A potently activated IκB kinase, c-Jun N-terminal kinase (JNK), Erk and Akt and enhanced NF-κB-dependent luciferase activity in primary human and rat HSCs. Serum amyloid A induced the transcription of MCP-1, RANTES and MMP9 in an NF-κB- and JNK-dependent manner. Blockade of NF-κB revealed cytotoxic effects of SAA in primary HSCs with signs of apoptosis such as caspase 3 and PARP cleavage and Annexin V staining. Serum amyloid A induced HSC proliferation, which depended on JNK, Erk and Akt activity. In primary hepatocytes, SAA also activated MAP kinases, but did not induce relevant cell death after NF-κB inhibition. In two models of hepatic fibrogenesis, CCl_4_ treatment and bile duct ligation, hepatic mRNA levels of SAA1 and SAA3 were strongly increased. In conclusion, SAA may modulate fibrogenic responses in the liver in a positive and negative fashion by inducing inflammation, proliferation and cell death in HSCs.

## Introduction

Serum amyloid A (SAA) is a 12.5 kd acute phase protein which is highly conserved among all vertebrate species [1–3]. Serum amyloid A has been shown to play a protective role during inflammation [4]. After infection or injury, SAA levels increase up to 1000-fold reaching serum concentrations of up to 80 μM in total. While the majority of SAA is found in association with high density lipoproteins, up to 15% of SAA exists in a lipid-free or lipid-poor form [5]. Human SAA1 and SAA2, and murine SAA1, SAA2 and SAA3 are the main acute phase SAA proteins and predominantly produced by hepatocytes, whereas SAA4 is constitutively expressed [6]. Hepatic acute-phase SAA production is stimulated by LPS and TNFα in a NF-κB dependent manner, and accounts for up to 2.5% of protein produced in inflamed liver in humans and up to 10% in other species. SAA has been suggested to play a role in inflammatory diseases such as atherosclerosis, rheumatoid arthritis and chronic inflammatory bowel disease [7–10]. Other studies propose functions for SAA in cholesterol transport [2, 3, 11]. Recently, it has been demonstrated that SAA may elicit cytokine and chemokine production, cell migration and upregulation of MMPs [6, 12–15]. On the molecular level, SAA has been shown to stimulate several proinflammatory and anti-apoptotic signaling pathways including NF-κB, C/EBP, JNK, Erk, Akt and p38 [10, 14–16]. Its role in liver injury and fibrogenesis is, however, yet ill-defined.

In this study, we investigate whether SAA may be involved in a potential crosstalk between hepatocytes as its major producing cell type and hepatic stellate cells (HSCs). HSCs are a pericyte-like cell population in the liver that normally store a large proportion of the body’s vitamin A. Following hepatic injury, HSCs undergo an activation process to become the predominant extracellular matrix producing cell population [17, 18]. Here we demonstrate that SAA levels are strongly elevated in 2 mouse models of hepatic fibrosis, and that SAA elicits inflammation, proliferation and apoptosis in HSCs suggesting SAA as a potential mediator of hepatocyte-HSC crosstalk in the injured liver.

## Experimental Procedures

### Cell isolation and culture

Primary HSCs were isolated by a 2-step collagenase perfusion from surgical specimens of healthy human livers (n = 3), from livers of male Sprague-Dawley rats (300–450 g, n = 20) or male Balb/c mice (n = 15) followed by Nycodenz (Nycodenz, Oslo, Norway) two-layer discontinuous density gradient centrifugation as described [19–22]. All tissues were obtained by qualified medical staff, with written donor consent and the approval of the Ethics Committee of Columbia University, according to the Declaration of Helsinki. Purity of human, rat and mouse HSC preparations was 88, 94 and 96%, respectively, as assessed by autofluorescence at day 2 after isolation. Hepatic stellate cells were cultured in DMEM containing 10% fetal bovine serum and standard antibiotics on uncoated plastic tissue culture dishes. Culture-activated human HSCs were used between passages 2 to 7. Rat and mouse HSCs were not passaged and considered culture-activated between day 7 and 14 after isolation. Primary skin fibroblasts were isolated from mouse from C57BL/6J wt, IL-1R knockout, TNF-R1 knockout and IL-1R, TNF-R1 double knockout mice by skin excision and culture in DMEM media plus 10% fetal bovine serum and antibiotics. Skin fibroblasts were used between passage 2 and 4. TRAF2- and RIP-1-knockout MEFs (a gift from Dr. Michael Karin) have been described previously [23]. The animals were sacrificed by a lethal dose of ketamine under anesthesia. All animals received humane care and all procedures were approved by the Columbia University Institutional Animal Care and Use Committee and the Commitee for Animal Studies in North Rhine-Westphalia (LANUV 84–02.05.20.11.249) and are in accordance with the requirements set by the National Institutes of Health and the German Protection of Animals Act.

### RNA isolation from HSCs and fibrotic liver and real time PCR

Hepatic fibrosis was induced by injecting Balb/c mice with 0.5 μl CCl_4_/g body weight intraperitoneally once per week for 8 weeks or by performing bile duct ligation as previously described [20]. Human cells were treated with recombinant human SAA (PeproTech, Rocky Hill, NJ) or recombinant human soluble Fas receptor (sFasR, PeproTech), murine cells were treated with recombinant human SAA or recombinant murine SAA, rmTNFα or rmIL-1β (all R&D Systems, Minneapolis, MN, as indicated) in the presence or absence of adenoviral GFP or IκBsr, or SP600125 (20 μM) or LY294002 (10 μM) as described above. Recombinant human SAA corresponds to the sequence of human SAA 1 isotype except for addition of an N-terminal Met and substitution of Asp for Asn at position 60 and substitution of His to Arg at position 71. RNA was extracted by homogenizing tissue or cells in Trizol according to the manufacturer’s instructions (Invitrogen, Carlsbad, CA). Reverse transcription was performed using random hexamer primers according to the manufacturer’s instructions (Amersham Biosciences, Piscataway, NJ). Real time PCR was performed for 40 cycles of 15s at 95°C and 60s at 60°C using an ABI 7000 sequence detection system as described [24]. Each sample was measured in duplicate and quantification was performed by comparing the C_t_ values of each sample to a standard curve. Probes and primers for human RANTES, human MCP-1, human IL-8, human MMP9, 18s, mouse SAA1, mouse SAA3 and mouse GAPDH were designed by ABI. RANTES, MCP-1, IL-8 and MMP9 levels were normalized to 18s, SAA1 and SAA3 levels were normalized to GAPDH and are expressed as fold induction compared to untreated control. PCR products were partly analyzed on a 1.5% agarose gel.

### ELISA for IL-8, MCP-1 and RANTES

Human HSCs were infected with AdGFP or AdIκBsr at a multiplicity of 500 particles/cell one day before treatment or pretreated with LY294002 (10 μM), SP600125 (20 μM) or DMSO (0.1%) for 30 minutes where indicated followed by treatment with rhSAA (5 μg/ml) for 18h. Levels of human IL-8, MCP-1 and RANTES were determined by sandwich ELISA (R&D Systems) as previously described [25].

### Western Blot Analysis

Protein extracts were run on 10–12% SDS acrylamide gels and transferred onto nitrocellulose as previously described [19–21]. Blots were incubated with anti-caspase-3, anti-PARP, anti-phospho-Erk, anti-phospho(S536)-p65, anti-phospho(S473)-Akt (all Cell Signaling Technology, Beverly, MA), anti-IκBα, anti-phospho(S63)-c-Jun (both Santa Cruz Biotechnology, Santa Cruz, CA), anti-α-smooth muscle actin (αSMA, Sigma-Aldrich, St. Louis, MO) overnight at 4°C. After incubation with secondary horseradish-peroxidase conjugated antibodies (Santa Cruz Biotechnology), the bands were visualized by the enhanced chemiluminescence light method (Amersham Biosciences) and exposed to X-omat film (Eastman Kodak Co., New Haven, CT) or a chemiluminescence imager (Image Station 2000R, Eastman Kodak Co.). Blots were reprobed with monoclonal anti-actin (MP Biomedicals) or GAPDH (Cell Signaling Technology) to demonstrate equal loading.

### Kinase assays

Human HSCs were serum starved for 12h followed by treatment with rhSAA or rhTNFα. IKK activity was determined by *in vitro* kinase assay as previously described [19, 26]. Briefly, 300 μg protein were immunoprecipitated with 2 μl anti-IKKγ (Santa Cruz) and washed three times. The kinase reaction was performed in 25 μl kinase buffer containing 0.5 μCi 32^P^-labeled ATP, 5 μM ATP and 80 μg/ml substrate (either GST-IκB (1–54), GST-p65 (354–551) or GST-p65 (354–551, 536 A) for 30 minutes at 30°C. Plasmids for GST-p65 substrates were a gift from H. Sakurai (Tanabe Seiyaku Co., LTD., Osaka, Japan). JNK activity was determined by an *in vitro* kinase assay as previously described [19]. Briefly, 25 μg of whole cell extract were precipitated with GST-c-Jun sepharose beads, washed three times and subjected to a kinase reaction for 30 minutes at 37°C. Subsequently the beads were boiled in Laemmli-buffer and run on a 10% SDS-acrylamide gel. The gels were stained with Coomassie blue to confirm equal substrate loading, dried, exposed to Kodak Biomax film and/or to a Phosphoimager (Molecular Dynamics, Sunnyvale, Ca) for quantification of the bands.

### NF-kB reporter assay

Human HSCs were infected with an adenovirus containing a NF-κB-driven luciferase using a multiplicity of infection of 250 [27]. 12h later, the supernatant was changed and cells were serum starved for 12h followed by treatment with rhSAA or rhTNFα for 6h. Luciferase activity was detected by luminescence (Becton Dickinson, Lincoln Park, NJ) on a Fluostar Optima platereader (BMG, Durham, NC) and normalized to protein content as determined by the Bradford method.

### [3H]-Thymidine Incorporation Assay

Serum-starved HSCs (0.4×10^5^/well) were incubated with rhSAA or PDGF-BB (Sigma) in the presence or absence of PD. 16h later, the cells were pulsed with 1 μCi/ml [^3^H]-thymidine (Amersham Biosciences) for 8h, followed by TCA-precipitation, lysis, and measurement in a scintillation counter.

### Determination of cell death

Activated rat HSCs were infected with AdIκBsr or AdGFP at 500 moi for 12h followed by serum starvation for 12h and rhSAA (5 μM) or rhTNFα (R&D Systems; 10 ng/ml) treatment for 8h. For some experiments, HSCs were co-cultured with FasL-expressing 3T3 fibroblasts or control 3T3 fibroblasts [28] at a HSC to 3T3 ratio of 1:5. Primary mouse hepatocytes were treated with rmSAA (5 μM) or rmTNFα (30 ng/ml, R&D Systems) with or without ActD (0,2 μg/ml, Sigma) after 12h of serum starvation. Cell death in HSCs or hepatocytes was measured by LDH release into the culture medium according to the manufacturer’s instructions (Roche, Indianapolis, IN). Caspase 3-cleavage and PARP cleavage were determined by western blot. Apoptosis was visualized by fluorescent microscopy of PI- and Annexin V-staining (Roche).

### Statistical analysis

All data represent the mean of 3 independent experiments ± SD, if not otherwise stated. For the determination of statistical significance, unpaired Student’s t-tests were performed using Prism (GraphPad, San Diego, CA). P values of <0.05 were considered to be statistically significant.

## Results

### SAA induces activation of IKK/NF-κB and JNK in primary HSCs

**Fig 1:**
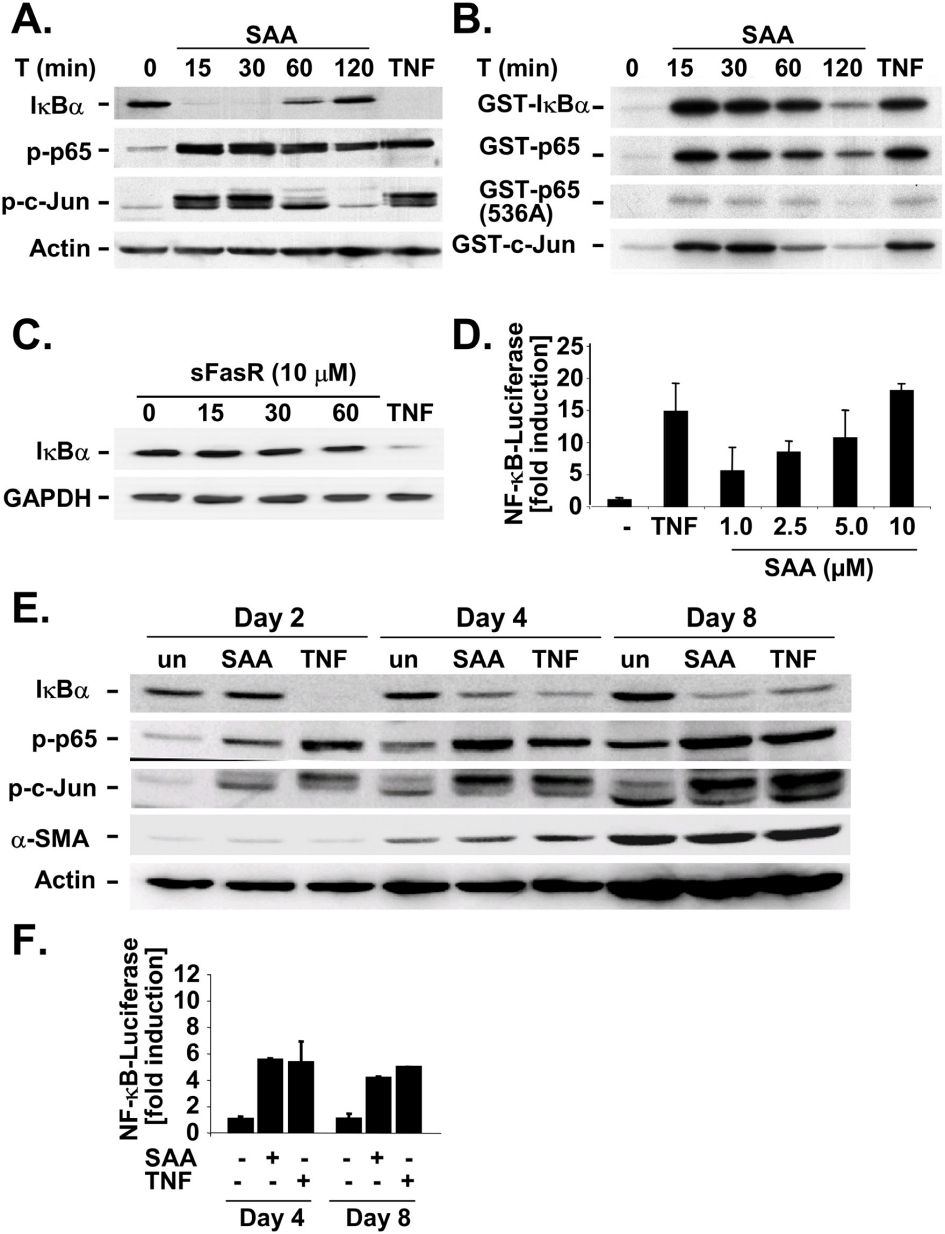
SAA induces IKK and JNK activation in HSCs. **A-B.** Activated human HSCs were treated with recombinant human SAA (5 μM) for the indicated times or with rhTNFα for 15 minutes. Extracts were subjected to western blot analysis using antibodies that recognize IκBα, phospho-p65 (S536), phospho-c-Jun or actin (**A**). Results were confirmed by *in vitro* kinase assay using GST-IκBα, GST-p65, GST-p65 (S536A) and GST-c-Jun substrates (**B**). **C.** Activated human HSCs were treated with recombinant human sFasR (10 μM) for the indicated times or with rhTNFα for 15 minutes. Extracts were analyzed by western blot using antibodies against IκBα or GAPDH. **D.** Activated human HSCs were infected with adenoviruses expressing NF-κB driven luciferase followed by treated with the indicated concentrations of SAA or with rhTNFα (10 ng/ml) for 6h. Lucerifase activity is expressed as fold induction in comparison to untreated control. **E.** Primary rat HSCs were activated for 2, 4 and 8 days and treated with SAA (5 μM) or rmTNFα (30 ng/ml) for 15 minutes. Extracts were subjected to western blot analysis using antibodies that recognize IκBα, phospho-p65 (S536), phospho-c-Jun or actin. **F.** Primary rat HSCs were activated for infected with adenoviruses expressing NF-κB driven luciferase at day 3 or day 7 after plating. 18 hours after infection, cells were treated with with SAA (5 μM) or rmTNFα (30 ng/ml) for 15 minutes. NF-κB driven luciferase was determined as described above.

SAA is secreted by hepatocytes and represents one of the most abundant proteins in the liver during the acute phase response [2, 3]. To assess whether SAA may influence the fibrogenic response in the liver, we determined its effect on proinflammatory and anti-apoptotic pathways in cultured HSC, which are believed to play an essential role in HSC activation and perpetuation [29, 30]. First, we measured activation of IKK and JNK, which are upstream activators of the NF-κB and the AP-1 pathways. Recombinant human SAA induced a strong activation of IKK as determined by western blot for its target IκBα, which was completely degraded after 15 and 30 minutes of stimulation (Fig 1A). Additionally, we observed a strong S536 phosphorylation of p65, a second target of IKK. SAA also strongly induced the JNK pathway as determined by western blotting for the phosphorylation of its target c-Jun (Fig 1A). These results were confirmed by an *in vitro*-kinase assay which showed potent activation of IKK towards GST-IκBα and GST-p65 and JNK toward GST-c-Jun with a maximum activation occurring after 15 minutes (Fig 1B). To rule out unspecific effects of SAA, e.g. by contaminants, we used human recombinant soluble Fas receptor, sFasR, as control and checked for IκBα degradation. In contrast to SAA and to TNFα as positive control, sFasR did not degrade IκBα (Fig 1C). Notably, SAA-induced IKK and JNK activation were as potent as TNFα-induced IKK activation (Fig 1A and 1B). These results were further confirmed by a reporter gene assay in which human HSCs were infected with an adenovirus containing 3xκB driven luciferase. SAA induced a dose-dependent increase in NF-κB-dependent luciferase activity and exceeded the effects of TNFα at a concentration of 10 μM (Fig 1D). Next we tested whether this effect also occurred in primary rat HSCs. Similar to the results obtained with human HSCs, we observed a potent activation of the JNK and IKK pathways culture-activated rat HSCs (day 4 and day 8), but only a weak activation in quiescent HSCs (day 2) (Fig 1E). In activated rat HSCs, SAA-induced NF-κB driven luciferase activity reached or even exceeded levels of those induced by TNFα (Fig 1F).

### SAA induces chemokine and MMP9 transcription through NF-κB and JNK

**Fig 2:**
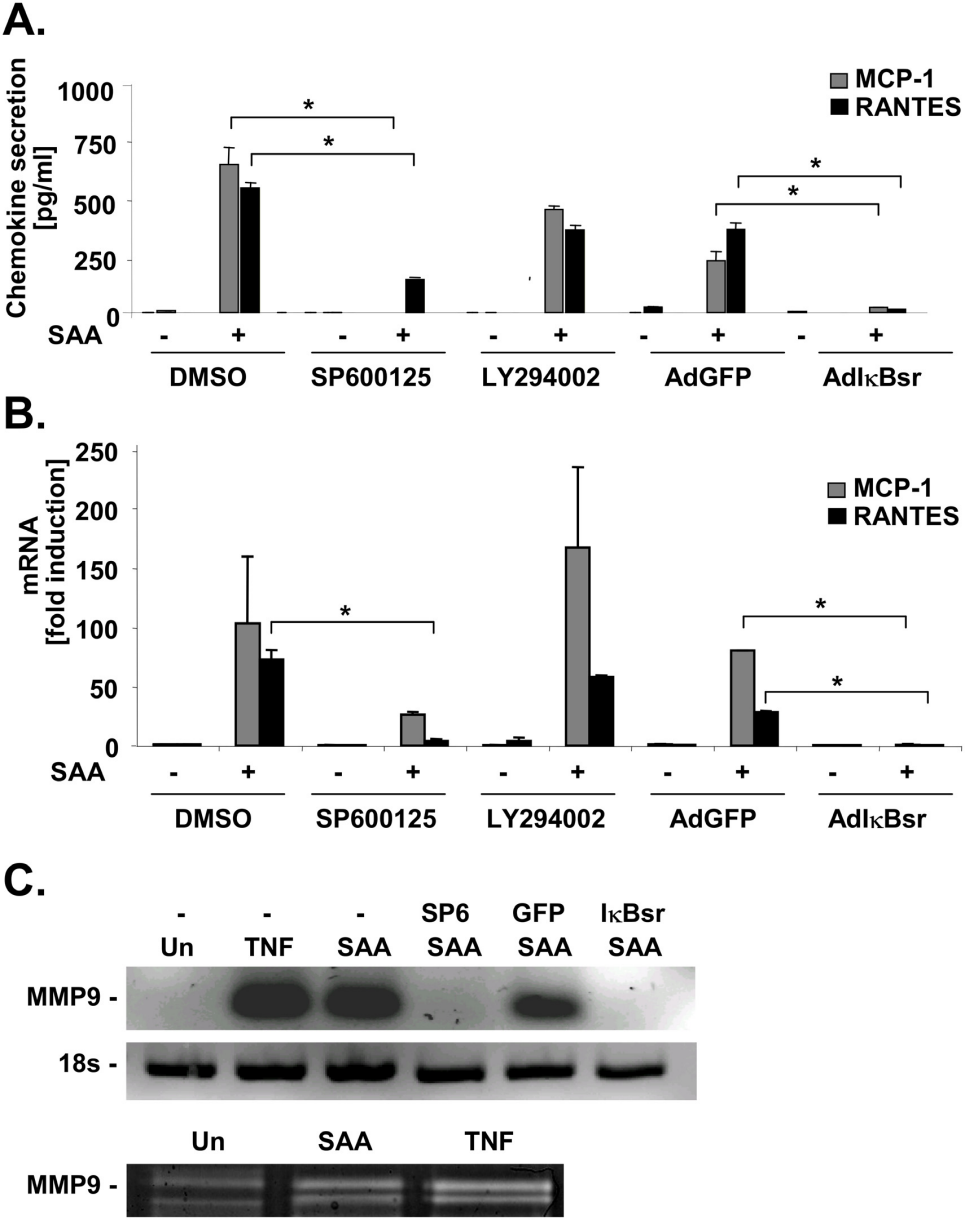
SAA induces chemokine and MMP 9 expression in HSCs in an NF-κB- and JNK-dependent manner in HSCs. Activated human HSCs were treated with rhSAA in the prescence or absence of JNK inhibitor SP600125 (20 μM), or after infection with adenoviruses expressing either IκBsr or GFP. **A.** 24 hours after rhSAA treatment, supernatants were analyzed for MCP-1, IL-8 and RANTES secretion by ELISA. **B-C.** 6 hours after rhSAA treatment, RNA was extracted and analyzed for mRNA levels of MCP-1, IL-8 and RANTES (**B**) or MMP9 (**C**) by quantitiative real time PCR (*p<0.05 vs. SP600125 treatment or IκBsr infection, resp.). **D.** 24 hours after SAA treatment, proteins were extracted and MMP9 activity was analyzed by zymography.

To test whether the increased activity of IKK after SAA stimulation resulted in an increased transcription of NF-κB dependent genes in HSCs, we measured the secretion of the NF-κB-dependent chemokines IL-8, MCP-1 and RANTES by ELISA. Recombinant human SAA strongly induced the secretion of all three chemokines (Fig 2A). To address the question whether the IKK and JNK pathways are responsible for driving the induction of these chemokines, we pretreated HSCs with pharmacological inhibitors of JNK and Akt, or infected them adenovirus expressing IκBsr, a potent inhibitor of the NF-κB pathway. Whereas inhibition of NFκB and JNK resulted in a complete or partial inhibition of chemokine secretion, respectively, we did not observe a significant inhibition of chemokine secretion with the PI-3 kinase inhibitor LY294002 (Fig 2A). Thus JNK and NF-κB, but not PI-3kinase/Akt are signaling pathways that mediate the upregulation of IL-8, MCP-1 and RANTES in human HSCs in response to SAA. Next we investigated whether NF-κB and JNK inhibition also reduce chemokine production at the mRNA level. Real time PCR measurements showed that IκBsr almost completely reduced the SAA-induced secretion of all 3 chemokines, whereas the JNK inhibitor SP600125 reduced chemokine secretion 50 to 80% (Fig 2B). Additionally, we also investigated whether SAA upregulated the levels MMP9 and MMP13, two crucial regulators of extracellular matrix and inflammation in the liver [31, 32]. Whereas we did not find detectable levels of MMP13 in the presence or absence of SAA (data not shown), we detected a strong induction of MMP9 after SAA treatment, which was blocked after incubation with either SP600125 or infection with AdIκBsr (Fig 2C, upper panel). The induction of MMP9 by SAA was confirmed by zymography which showed an increased MMP9 activity after SAA or TNFα (Fig 2C panel), yet to a much lower extent than the induction found at the mRNA level.

### SAA activates Erk, Akt and JNK to induce HSC proliferation

**Fig 3:**
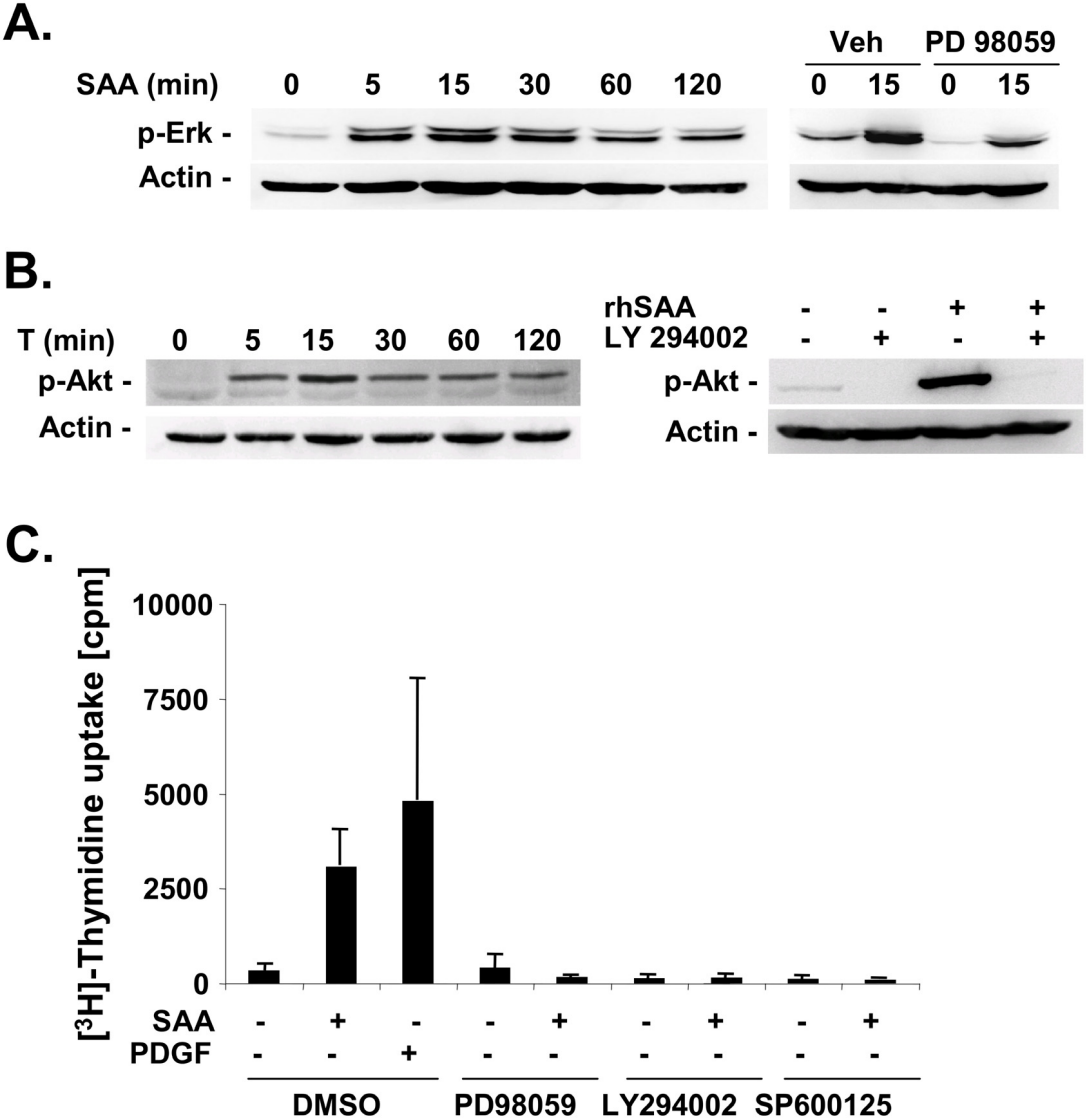
SAA induces HSC proliferation in an Erk-, JNK- and Akt-dependent manner. **A-B.** Activated rat HSCs were treated with rhSAA (5 μM) for the indicated times. Phosphorylation of Erk at threonine 202 and tyrosine 204 (**A**), and Akt at serine 473 (**B**) was determined by western blot analysis in the absence or presence of MEK inhibitor PD98059 or PI-3 kinase inhibitor LY294002, respectively. **C.** Serum starved activated rat HSCs were pretreated with PD98059 (5 μM), LY294002 (10 μM) or SP600125 (20 μM) followed by stimulation with rhSAA for 24 hours. 18 hours after stimulation cells were pulsed with 1 uCi/ml 3H-thymidine followed by TCA precipitation and measurement in a scinitillation counter.

Next we determined whether SAA activated Erk and Akt in HSCs as previously reported in other cell types [10]. Both Akt and Erk have been implicated in HSC proliferation [33, 34]. Erk phosphorylation was strongly activated by SAA with a maximum between 5 and 30 minutes after stimulation, and inhibited by MEK inhibitor PD98059 (Fig 3A). We observed a strong activation of Akt after SAA as apparent by S473 phosphorylation already after 5 minutes of stimulation which lasted for up to two hours (Fig 3B). SAA-induced Akt S473 phosphorylation was completely blocked by the PI-3 kinase inhibitor LY294002 demonstrating that SAA-induced Akt activation is mediated by its upstream activator PI-3 kinase. To determine whether activation of Akt and Erk in response to SAA had any effect on HSC proliferation, we investigated SAA-induced [^3^H]-thymidine uptake in the presence or absence of PI-3 kinase and MEK inhibitors. SAA-induced a strong induction of [^3^H]-thymidine uptake which was almost 60% of that induced by 5 nM PDGF (Fig 3C). Pharmacological inhibition of PI-3 kinase and MEK as well as of JNK completely blocked SAA-induced [^3^H]-thymidine uptake suggesting that activation of these pathways is responsible for the proliferative effects of SAA (Fig 3C).

### SAA induces apoptosis after NF-κB inhibition in primary HSCs, but not in hepatocytes

**Fig 4:**
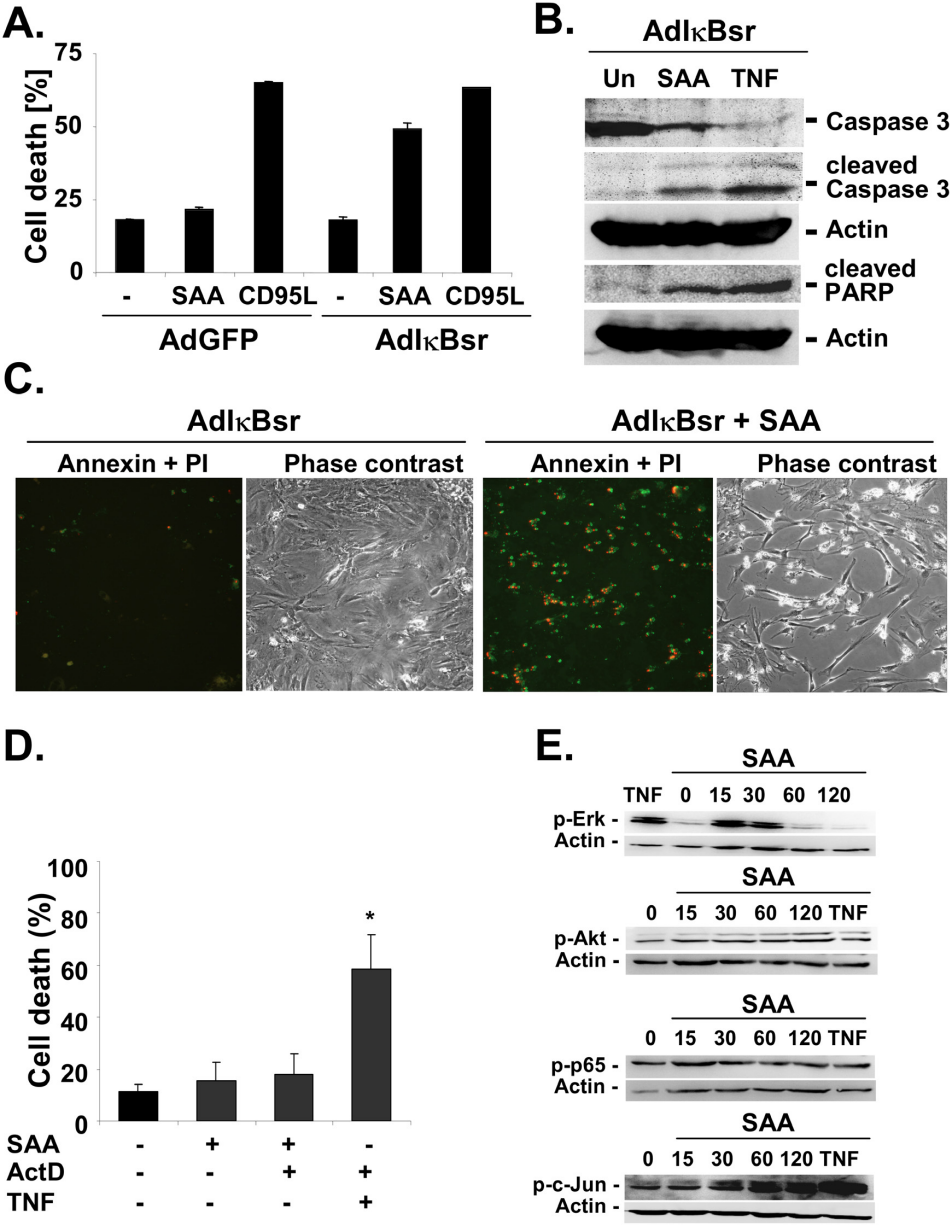
SAA induces apoptosis in HSCs after NF-κB inhibition. Activated rat HSCs were infected with adenoviruses expressing either GFP or IκBsr. 24 hours later, HSCs were treated with rhSAA (5 μM). **A.** After 18h or treatment, cell death was determined by LDH activity assay. **B.** Caspase 3 and PARP cleavage were determined by western blot analysis after 8 hours of rmSAA1 treatment. **C.** After 8 hours of rhSAA treatment, cells were stained with Annexin V (green) and propidium iodide (PI, red) to visualize apoptosis and necrosis. **D.** Primary mouse hepatocytes were serum-starved for 12h and treated with rmSAA (5 μM), ActD (0.2 μg/ml) or rmTNFα (30 ng/ml) for 18. Cell death was determined by LDH activity assay (*p<0.05 vs. untreated vehicle control). **E.** Primary mouse hepatocytes were treated with rmSAA (5 μM) for the indicated times and rmTNFα (30 ng/ml) for 120 min. Phosphorylation of Erk, Akt, p65 and c-Jun was determined by western blot analysis.

Many of the signaling pathways induced by SAA resembled those activated by TNF. Therefore, we next investigated whether SAA induces apoptosis in HSCs. Since human HSCs are highly resistant to TNFα-induced cell death [35], we investigated SAA-induced cell death in primary unpassaged rat HSCs which are susceptible to TNFα-induced apoptosis after NF-κB inhibition [36]. As expected, we did not detect an increase in cell death in primary HSCs that were treated by SAA alone. However, when NF-κB activation was blocked by IκBsr in primary rat HSCs, we observed almost 50% cell death after 24h of SAA treatment (Fig 4A). To determine whether cell death was apoptotic, we checked for the presence of cleaved caspase 3 and the 89kd PARP cleavage fragment, two hallmarks of apoptosis. After 8h of SAA treatment, we detected the occurrence of caspase-3 cleavage products at 17kd and 19kd as well as the 89kd PARP cleavage product (Fig 4B). To confirm these results, we stained HSC with AnnexinV and propidium iodide. After 8h of SAA, the majority of IκBsr-expressing cells were AnnexinV-positive indicating the onset of apoptotic cell death (Fig 4C). Interestingly, SAA treatment alone or after inhibition of NFκB activation by ActD did not cause cell death in primary mouse hepatocytes (Fig 4D), indicating a possible selective induction of cell death in HSCs and hepatocytes by SAA during hepatic injury and fibrogenesis. However, SAA treatment of hepatocytes also led to potent phosphorylation of Erk, and c-Jun and to a lesser extent of Akt and p65 (Fig 4E).

### SAA does not signal through TNF-R1, IL-1R or TLR4

**Fig 5:**
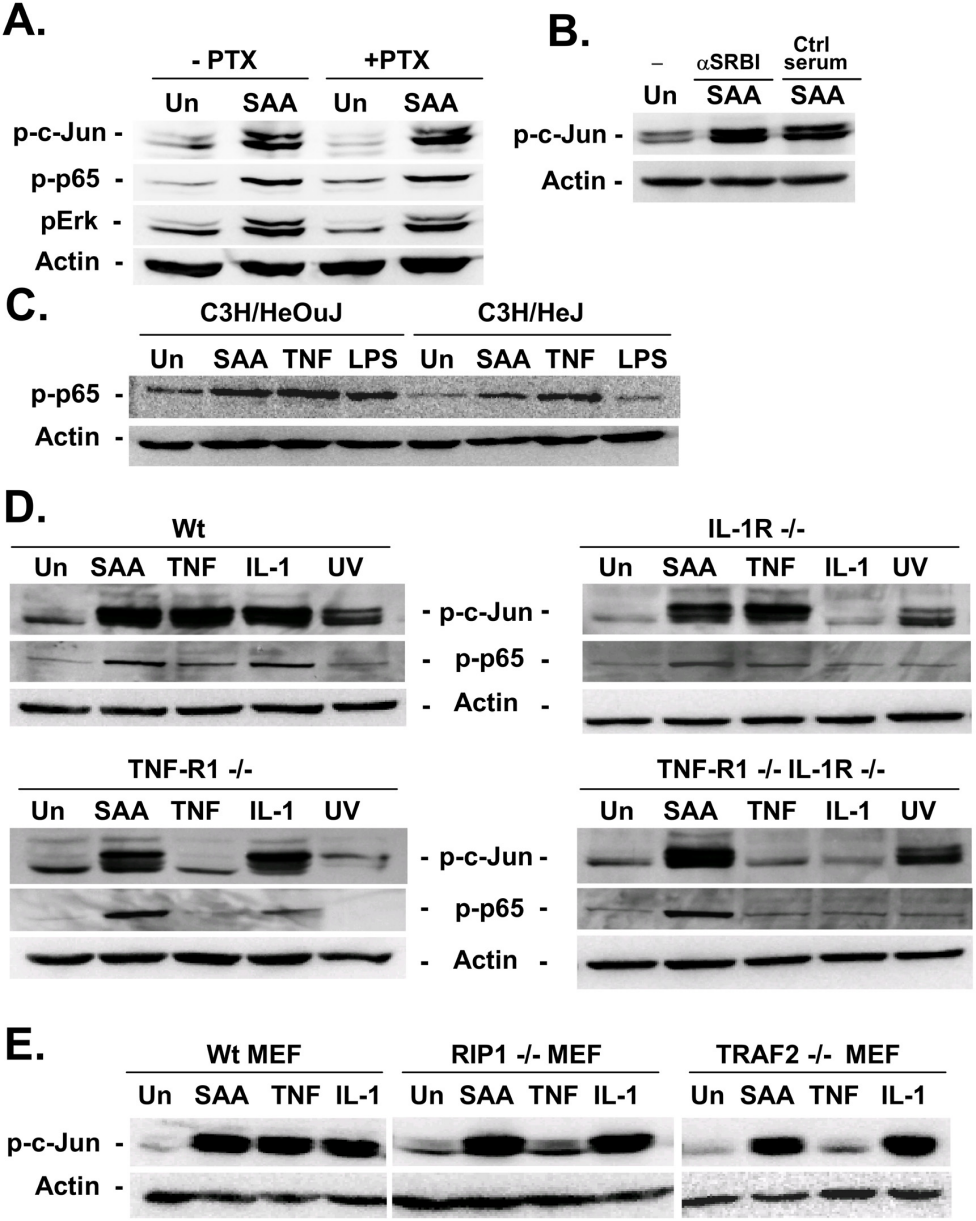
SAA does not signal in HSCs through TLR4, IL-1R, TNF-R1 or SR-BI. **A.** Activated human HSCs were pretreated with pertussis toxin followed by treatment with rhSAA (5 μM). Activation of JNK, IKK and Erk pathways was analyzed by immunoblot for p-c-Jun, p-p65 and pErk. **B.** Activated human HSCs were pretreated with SR-BI blocking antibody or control antibody followed by rhSAA (5 μM) stimulation. JNK activation was analyzed by western blot using phospho-specific antibodies for its substrate c-Jun. **C.** Skin fibroblasts from TLR4-sufficient C3H/HeOuJ and TRL4-deficient C3H/HeJ mice were stimulated with rhSAA (5 mM), rmTNFα (30 ng/ml) or LPS (100 nM) followed by immunoblot for p-p65. **D.** Skin fibroblasts from wild-type mice, TNFRI-knockout mice, IL-1 receptor knockout mice and from TNFRI and IL-1 receptor double knockout mice were treated with rhSAA (5 μM), rmIL-1β (5 ng/ml), rmTNFα (30 ng/ml) or irradiated with UV (100 J/m^2^) Activation of IKK and JNK was determined by western blot analysis for their phosphorylation of their targets p65 and c-Jun, respectively. E. Mouse embryonic fibroblasts from wild type mice, TRAF-2 deficient mice and RIP-1 deficient mice were treated rhSAA (5 μM), rmIL-1β (5 ng/ml), rmTNFa (30 ng/ml) or irradiated with UV (20 J/m^2^). Activation of IKK and JNK was determined by western blot analysis for their phosphorylation of their targets p65 and c-Jun, respectively.

SAA signaling is not well defined and several receptors including formyl peptide receptor like 1 (FPRL1) and SR-BI have been proposed as receptors for SAA [6]. We did not see any effects on SAA-induced JNK activation when we blocked FPRL1 signaling by pertussis toxin or SR-BI by a blocking antibody (Fig 5A). Our previous observation showed that SAA activated pathways that are induced in similar manner by TNFα, and to some extent by LPS and IL-1β (Figs 1–4). To investigate whether SAA-induced signals may be due to minute contamination with LPS, we first investigated SAA-induced p65 phosphorylation in TLR4-deficient fibroblasts. LPS did not induce p65 phosphorylation in these cells whereas TNFα and SAA induced p65 phosphorylation effectively excluding that SAA-mediated effects were due to LPS contamination (Fig 5B). To exclude involvement of TNF-R1 and IL-1R, we treated fibroblasts deficient in TNF-R1 and/or IL-1R with SAA. Whereas TNFα and IL-1β signaling was completely abolished in these cells, SAA-induced c-Jun phosphorylation as well as UV-induced c-Jun phosphorylation (serving as positive control) was present demonstrating that TNF-R1 and IL-1R are not involved in the SAA pathway (Fig 5C). Similarly, we found an induction of NF-κB p65 phosphorylation in IL-1R- and/or TNFR1-deficient cells. To explore whether SAA-induced pathways are similar to the TNF pathway downstream of the TNF receptor at the level of adapter molecules, we treated RIP1- and TRAF2-deficient MEFs with SAA, TNFα and IL-1β. Whereas TNFα signaling was completely blocked in RIP1- and TRAF2-deficient cells, SAA and IL-1β induced a strong activation of the JNK pathway (Fig 5D). In conclusion, our results demonstrate that SAA signals through an unknown receptor in fibroblasts and this pathway is independent of TLR4, IL-1R, TNFR1, TRAF2 and RIP1, and not sensitive to inhibition by pertussis toxin.

### Hepatic SAA1 and SAA3 levels are increased during fibrogenesis

**Fig 6:**
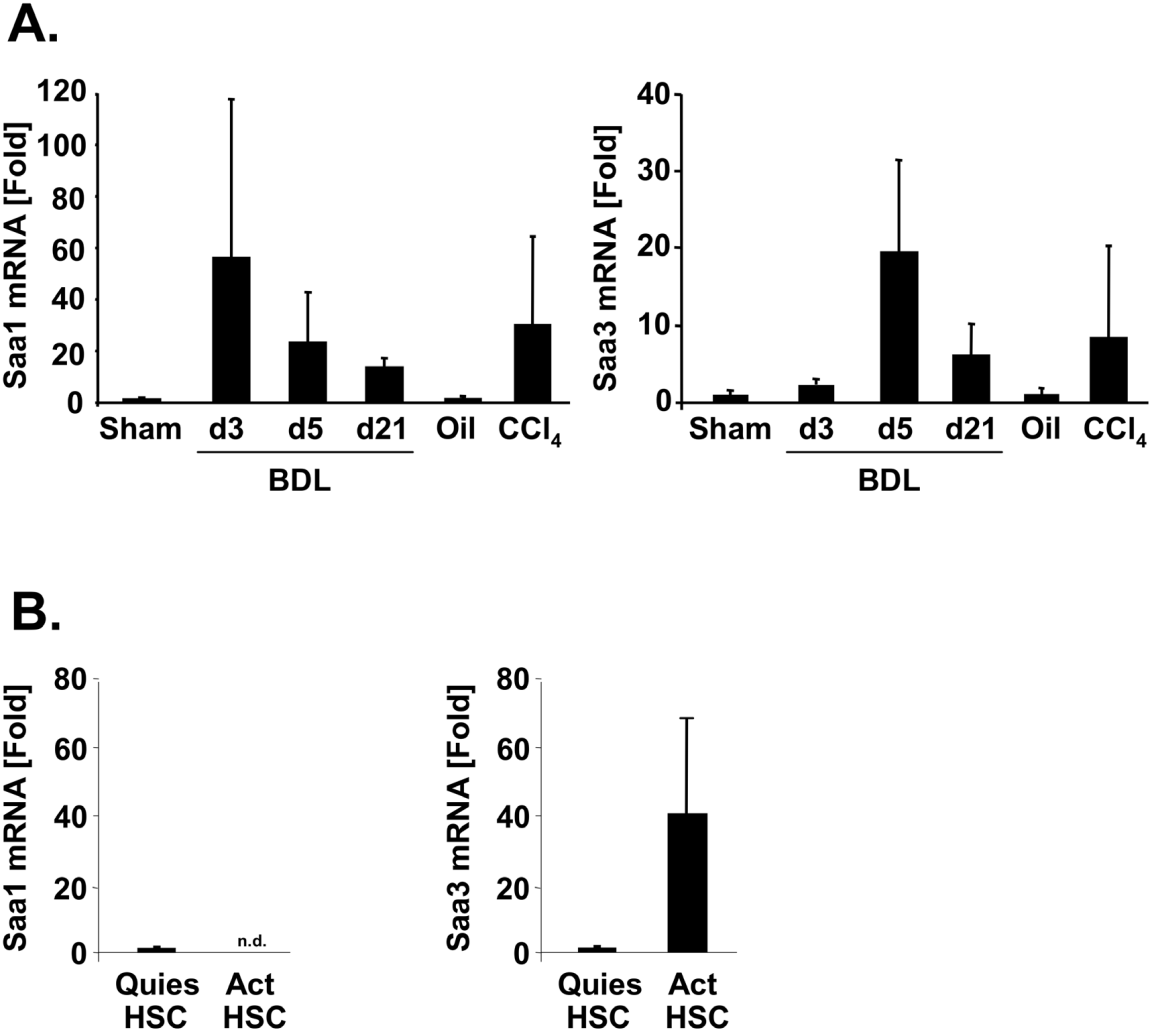
SAA1 and SAA3 levels are elevated during hepatic fibrogenesis. **A.** Hepatic fibrosis was induced in 8 week old Balb/c mice by bile duct ligation (n = 3 mice per time point) or by treatment with CCl_4_ (8 injections of 0.5 μl CCl_4_/g body weight, dissolved in olive oil, every 3 days, n = 4 mice/group). Following RNA extraction and reverse transcription, mRNA levels of SAA1 and SAA3 were determined by quantitative real time PCR. Levels are expressed as fold-induction in comparison to sham or oil control mice after normalization to 18s. **B.** RNA was isolated from quiescent mouse HSCs (18 hours after plating, n = 3 independent isolations) or activated mouse HSCs (5 days after plating, n = 3 independent isolations). mRNA levels of SAA1 and SAA3 were determined by quantitative real time PCR. Levels are expressed as fold-induction in comparison to quiescent HSCs after normalization to 18s.

To determine whether SAA levels are increased during hepatic fibrogenesis and may thus influence HSCs *in vivo*, we measured SAA mRNA levels in 2 models of hepatic fibrogenesis by quantitative real time PCR. While SAA1 is the main acute phase SAA produced by hepatocytes, SAA3 is predominantly produced by macrophages during the acute phase response. The levels of SAA1 and SAA3 mRNA were induced 28-fold and 8-fold, respectively, in mice after 8 injections of CCl_4_ (Fig 6A). In mice that underwent bile duct ligation to induce fibrogenesis, we measured a 55-fold and 2-fold increase in SAA1 and SAA3 mRNA three days after bile duct ligation, respectively (Fig 6A). Five days after bile duct ligation, SAA1 and SAA3 mRNA were increased 23-fold and 19-fold, respectively. Three weeks after bile duct ligation the mRNA levels of SAA1 and SAA3 still remained elevated, but were significantly lower than at day 5. In freshly isolated quiescent HSCs, we found only low levels of SAA1 mRNA in quiescent HSCs, which were 76000 times lower than those of hepatocytes and attributable to the small degree of hepatocyte contamination (data not shown). After 5 days of culture-activation, the levels of SAA1 became undetectable in HSCs consistent with the disappearance of hepatocyte contamination in culture (Fig 6b, left panel). In contrast, the levels of SAA3 were markedly upregulated during HSC culture-activation (Fig 6b, right panel). Thus, HSCs are likely to be a target of SAA1 and a source of SAA3 in the liver.

## Discussion

Although SAA is one of the most abundantly expressed hepatic proteins during infection and inflammatory conditions, its functions remain incompletely understood. The present study provides evidence that SAA modulates fibrogenic responses in the liver by inducing proliferation and inflammation in HSCs under some conditions and by promoting HSC cell death under other conditions.

Previous studies have reported that SAA exerts cytokine-like properties in many cell types. Consistent with these observations, we detected the activation of a large number of proinflammatory and anti-apoptotic signaling pathways in HSCs and hepatocytes. Surprisingly, the effects of SAA on mouse and human HSCs on pathways such as NF-κB and JNK were as strong as those exerted by TNFα and IL-1β, one of the most potent proinflammatory cytokines in HSCs and other cell types [19, 25]. Stimulation with SAA led to a dramatic increase in the production of chemokines such as IL-8, MCP-1 and RANTES. To exclude that signals mediated by SAA were due to a contamination with LPS or by TLR-agonistic activity of SAA as suggested by recent publications [37–39], we investigated proinflammatory signaling pathways in cells from TLR4-mutant mice. We found no difference between TLR4 wild-type and TLR4-mutant cells effectively excluding LPS as a contaminating agonist. Additionally, we investigated whether SAA might directly or indirectly activate receptors for TNFα or IL-1β. Again, there was no significant inhibition of SAA signals in cells deficient for TNFR1, IL-1 receptor or both receptors. Previous reports have suggested several receptors to mediate SAA effects, among these FLRP-1 and SR-BI [11, 14]. However, we could not find a role for these receptors in mediating proinflammatory effects of SAA in primary HSCs. Inflammation is tightly associated with hepatic fibrogenesis and considered a key event that promotes the activation of HSCs [29, 40, 41]. Increased production of chemokines by HSCs allows HSCs to recruit cell populations such as Kupffer cells whose presence is required for HSCs to fully activate [42, 43] [44]. Moreover, secretion of chemokines is thought to exert autocrine and paracrine effects on HSCs further leading to activation and to the recruitment of additional HSCs to the site of injury [45, 46]. Thus, SAA-mediated chemokine secretion in activated HSCs is likely to contribute to fibrogenesis and may represent a novel link between hepatocytes and HSCs in the injured liver.

Induction of HSC proliferation represents a second mechanism by which SAA may enhance fibrogenesis in the liver. Following activation, HSCs become highly proliferative and thus expand to a pool of activated HSCs to enhance fibrogenic responses. Accordingly, blockade of PDGF, the most potent mitogen for HSCs, significantly reduces hepatic fibrogenesis. We found that the pro-proliferative effects of SAA were weaker than those exerted by PDGF, yet stronger than those reported other agonists including insulin, leptin and chemokines such as MCP-1 and RANTES [25, 45, 47, 48]. Our results are similar to studies that show SAA-induced proliferation in human fibroblast-like synoviocytes, and suggest that SAA may exert similar functions in chronic inflammatory conditions of different organs.

Blockade of SAA-induced signaling pathways revealed specific functions in regards to proliferation and inflammation. While NF-κB and JNK activation were required for SAA-mediated proinflammatory gene expression and MMP9 expression, JNK, Akt and Erk were essential for SAA-induced proliferation. Blocking NF-κB was more efficient in reducing chemokine expession than blocking JNK. These findings are similar to previous results in TNFα- and IL-1β induced chemokine regulation in HSCs suggesting that NF-κB plays a more prominent role in regulating these genes than the JNK/AP-1 pathway [25, 49]. Previous studies have demonstrated that both Erk and Akt are required for HSC proliferation. Thus, our study provides evidence that SAA utilizes well-characterized pathways to induce inflammatory gene expression and proliferation in HSCs.

Amyloids are believed to be responsible for organ damage in diseases such as systemic amyloidosis and Alzheimer’s disease. Not only has SAA been shown to induce lysis of bacterial cells by forming ion-channels in lipid bilayer membranes [50], but has been reported to prevent cell death in eukaryotic cells [51, 52]. One study reported induction of nuclear changes consistent with apoptotic cell death after SAA treatment, but this study did not specify the type of SAA that was used for cell death induction nor did it provide quantification of cell death [53]. Here we show for the first time that NF-κB can act as a switch between SAA-induced cell death and proliferation. Similar to TNFα, SAA induced cell death in HSCs only in the absence of NF-κB activity. Thus, it seems that the anti-apoptotic effects of NF-κB regulated genes override proapopototic signals after SAA stimulation. SAA-mediated cell death was apoptotic as demonstrated by the appearance of caspase 3- and PARP-cleavage products and Annexin V membrane staining. This novel function of SAA suggests that in a fashion similar to TNF, SAA may act as double-edged sword in chronic inflammatory states: Induction of inflammation under most conditions, but induction of cell death when NF-κB dependent genes cannot be transcribed.

In view of the abundance of SAA in the injured liver and its upregulation not only during liver injury but many other infectious and inflammatory conditions, it is unlikely that SAA is a primary stimulator of HSC activation and inflammation. This notion is further supported by our finding that SAA did not induce IκBα degradation in quiescent (day 2) HSCs and only weak phosphorylation of the JNK target c-Jun. We therefore suggest that SAA modulates inflammatory and proliferative response in HSCs after they have initiated the activation process. SAA plays a similar role in other chronic inflammatory conditions and stimulates wound healing response in atherosclerosis and rheumatoid arthritis [7, 8]. While SAA is likely to promote HSC activation under many circumstances, the induction of cell death after inhibition of NF-κB suggest that SAA can also exert opposite effects. When the underlying liver disease is eradicated and liver injury ceases, activated HSCs are either eliminated by induction of cell death or by deactivation [54–57]. Recently, it has been shown that inhibition of NF-κB activation during hepatic fibrogenesis is associated with an increase in HSC cell death and accelerates recovery from experimental liver fibrosis [30]. It is conceivable that SAA may contribute to the increased rate of HSC apoptosis in mice treated with NF-κB inhibitors such as sulfasalazine. Moreover, hepatocytes were not effectively killed by SAA even after NF-κB inhibition, suggesting that abundant SAA production during liver injury and fibrogenesis may contribute to a controlled clearance of activated HSCs thus leading to containment of an excessive wound healing response.

In conclusion, our study suggests that SAA represents a novel cytokine-like molecule that may mediate a crosstalk between hepatocytes and HSCs in the injured liver. Future studies need to define its receptor target and to confirm the potential pro- and antifibrogenic effects of SAA during liver fibrosis induction and resolution for potential therapeutic exploitation.
